# Plasma vaspin levels and clinical outcome in incident peritoneal dialysis patients

**DOI:** 10.1186/s12882-023-03259-2

**Published:** 2023-07-12

**Authors:** Win Hlaing Than, Gordon Chun-Kau Chan, Bonnie Ching-Ha Kwan, Ka-Bik Lai, Ronald Cheong-Kin Chan, Jeromy Yuen Chun Teoh, Jack Kit-Chung Ng, Winston Wing-Shing Fung, Kai-Ming Chow, Phyllis Mei-Shan Cheng, Philip Kam-Tao Li, Cheuk-Chun Szeto

**Affiliations:** 1 Carol & Richard Yu Peritoneal Dialysis Research Centre, Department of Medicine & Therapeutics, Prince of Wales Hospital, The Chinese University of Hong Kong, Hong Kong Shatin, NT, China; 2 Department of Medicine & Therapeutics, Prince of Wales Hospital, Hong Kong Shatin, NT, China; 3 Department of Anatomical & Cellular Pathology, The Chinese University of Hong Kong, Hong Kong, China; 4 Department of Surgery, The Chinese University of Hong Kong, Hong Kong, China; 5 Li Ka Shing Institute of Health Sciences (LiHS), The Chinese University of Hong Kong, Hong Kong, China

**Keywords:** Renal failure, Atherosclerosis, Metabolic syndrome

## Abstract

**Background:**

Vaspin is an adipokine that regulates glucose and lipid metabolism. Plasma vaspin level is increased in chronic kidney disease but decreased in hemodialysis patients. However, plasma vaspin level in peritoneal dialysis (PD) patients, as well as its prognostic role, has not been studied.

**Methods:**

We recruited 146 incident PD patients. Their baseline plasma vaspin levels, body anthropometry, the profile of insulin resistance, bioimpedance spectroscopy parameters, dialysis adequacy, and nutritional indices were measured. They were followed for up to 5 years for survival analysis.

**Results:**

The average age was 58.4 ± 11.8 years; 96 patients (65.8%) were men, and 90 (61.6%) had diabetes. The median vaspin level was 0.18 ng/dL (interquartile range [IQR] 0.11 to 0.30 ng/dL). Plasma vaspin level did not have a significant correlation with adipose tissue mass or baseline insulin level. However, plasma vaspin level had a modest correlation with the change in insulin resistance, as represented by the HOMA-IR index, in non-diabetic patients (*r* = -0.358, *p* = 0.048). Although the plasma vaspin level quartile did not have a significant association with patient survival in the entire cohort, it had a significant interaction with diabetic status (*p* < 0.001). In nondiabetic patients, plasma vaspin level quartile was an independent predictor of patient survival after adjusting for confounding clinical factors (adjusted hazard ratio 2.038, 95% confidence interval 1.191–3.487, *p* = 0.009), while the result for diabetic patients was not significant.

**Conclusions:**

Plasma vaspin level quartile had a significant association with patient survival in non-diabetic PD patients. Baseline plasma vaspin level also had a modest inverse correlation with the subsequent change in the severity of insulin resistance, but the exact biological role of vaspin deserves further studies.

**Supplementary Information:**

The online version contains supplementary material available at 10.1186/s12882-023-03259-2.

## Introduction

Peritoneal dialysis (PD) is a life-saving renal replacement therapy [1, 2]. However, there is a growing concern over the increased risk of cardiovascular disease (CVD) in PD patients. Traditional CVD risk factors are highly prevalent among PD patients, but there is now research focusing on the role of non-traditional CVD risk factors in PD [3, 4]. Despite the distinct advantages of PD, such as being home-based self-care, having simple equipment, and minimizing staff costs [5–7], the risk of CVD deserves specific attention in PD patients.

Obesity has become increasingly prevalent in the general public [8, 9] and individuals with newly started on PD [10, 11]. Moreover, it has been identified as a potential risk factor for mortality in PD patients [12, 13]. Due to the risk of inadequate dialysis [14] as well as its connection to insulin resistance and the onset of diabetes [10], obesity can have a negative effect on the outcome of PD patients. In recent years, research has revealed that adipose tissue acts as an endocrine organ, releasing a number of adipokines with far-reaching effects [15, 16], among which vaspin has several remarkable attributes that require further investigation.

Vaspin, also called SRPINA12, is a serine protease inhibitor secreted by adipose tissue [17]. Physiological studies showed that vaspin is related to the development of insulin resistance, obesity, and inflammation [17, 18]. Serum vaspin levels correlate with metabolic and renal parameters [19] and predict future cardiovascular events in patients with chest pain [20]. In patients with chronic kidney disease (CKD), plasma vaspin level is increased before dialysis [21] but decreased in the hemodialysis population [22]. However, the plasma vaspin level in PD patients, as well as its prognostic role, has not been evaluated. In the present study, we examined the relation between plasma vaspin levels and body-built, insulin sensitivity, mortality, and morbidity in incident PD patients.

## Patients and methods

### Overall design

This is a retrospective analysis of a prospective observational study was approved by the Joint Chinese University of Hong Kong – New Territories East Cluster Clinical Research Ethics Committee (approval number CREC-2008.554). The study recruited all consecutive new adult PD patients in our center from January 2011 to December 2013. Patients who were planned to have elective living donor transplants or transferred to other renal centers within 6 months were excluded. All study procedures were in compliance with the Declaration of Helsinki. We recruited 146 consecutive adult incident PD patients in our center. After written informed consent, and around 4 to 6 weeks after the patients were stable on PD, we performed routine biochemical tests, peritoneal transport study, anthropometric measurement, dialysis adequacy and nutritional status assessment, multi-frequency bioimpedance spectroscopy study, and arterial pulse wave velocity study. Plasma samples were collected at the same time for vaspin levels measurement. Charlson’s comorbidity index (CCI) was computed as previously described [23].

### Plasma vaspin level

Plasma vaspin level was measured by a commercially available ELISA kit (Vaspin Human ELISA Kit, BioVendor, Brno, Czech Republic), following the manufacturer’s instructions. All assays were performed in duplicate. The detection limit of Vaspin was 0.01 ng/ ml; the inter-assay coefficient of variation was 5.8%.

### Study of peritoneal transport

The traditional peritoneal equilibration test (PET) was performed 4 to 6 weeks after the patients had stabilized on PD, according to the method by Twardowski [24]. The dialysate-to-plasma creatinine ratio (D/P) was computed after adjusting for the influence of glucose. The mass transfer area coefficients (MTAC) of creatinine normalized for body surface area (BSA) were then obtained by applying the standard formula [25]. The BSA was calculated based on the formula described by Gehan and George [26].

### Anthropometric measurements

On the same day of PET, we also recorded anthropometric measures including body weight, body mass index (BMI), waist and hip circumference, mid-arm circumference, triceps and subscapular skinfold thickness. In addition, the waist-hip ratio (WHR) was computed. BMI was determined by the standard formula and further categorized according to the Asia Pacific version of the WHO classification [27, 28]. According to this system, BMI < 18 kg/m2 is underweight; 18 to 22.9 is normal weight; 23 to 24.9 is marginal overweight; 25 to 29.9 is overweight; and greater than 30 is obesity [27, 28]. Lastly, the conicity index at baseline was determined to quantify the degree of central obesity [29].

$$conicity\ index= \frac{waist\ circumference \left(m\right)}{0.109 \times \sqrt{\frac{body\ weight \left(kg\right)}{height \left(m\right)}}}$$

### Dialysis adequacy and nutritional status

The procedure for assessing dialysis adequacy had been detailed previously [30]. This involved collecting 24-hour urine and dialysate samples for the purpose of calculating the total Kt/V. Residual glomerular filtration rate (GFR) was calculated by averaging the 24-hour urea and creatinine clearance levels [31]. Patients’ nutrition status was determined by their serum albumin levels, Subjective Global Assessment (SGA) score, Comprehensive Malnutrition–Inflammation Score (MIS), normalized protein nitrogen appearance (NPNA), and fat-free edema-free body mass (FEBM). The 4-item 7-point SGA scoring system that has been validated in peritoneal dialysis (PD) patients was employed [32]. The MIS rating incorporated 10 factors, each assessed from 0 to 3, with a highest score of 30 achievable [33]. NPNA was calculated using the modified Bergstrom’s formula [34]. FEBM was calculated using the creatinine kinetic method following the Forbes and Brunining formula [35], and reported as a percentage of their ideal body weight.

### Multi-frequency bio-impedance spectroscopy study

We used the multi-frequency device (Body Composition Monitor, Fresenius Medical Care, Germany) as described previously [36, 37]. In this study, we analyzed the data on lean tissue mass (LTM), adipose tissue mass (ATM), the volume of over-hydration (OH), and the extracellular-to-intracellular volume (E: I) ratio.

### Arterial pulse wave velocity study

Arterial pulse wave velocity (PWV) was measured by an automatic computerized recorder and analyzed using the Complior @ SP program (Artech Medical, France) by the method described previously [38]. In the present report, we computed the carotid-radial and carotid-femoral PWV.

### Glucose metabolism and systemic inflammation

Fasting glucose levels were measured, after overnight fast and omissing of PD, one month after patients had a stable PD regimen and were classified according to the American Diabetes Association (ADA) standards [39]. In addition, the serum C-peptide, insulin level, hemoglobin A1c level and serum C-reactive protein (CRP) level were measured at the beginning of the PD program and then again two years later. The assessment of insulin resistance was done using the HOMA-IR index [40]:

$$\mathrm{HOMA}-\mathrm{IR}\;=\;\mathrm{fasting}\;\mathrm{glucose}\;\left(\mathrm{in}\;\mathrm{mmol}/\mathrm l\right)\;\times\;\mathrm{fasting}\;\mathrm{insulin}\;\left(\mathrm{in}\;\upmu\mathrm{U}/\mathrm{ml}\right)\;/\;22.5$$

Similarly, the beta cell function was represented by the HOMA-2B index [40]:

$$\mathrm{HOMA}-2\mathrm B\;=\;20\;\times\;\mathrm{fasting}\;\mathrm{insulin}\;\left(\mathrm{in}\;\upmu\mathrm{U}/\mathrm{ml}\right)\;/\;\mathrm{fasting}\;\mathrm{glucose}\;\left(\mathrm{in}\;\mathrm{mmol}/\mathrm{ml}\right)\;-\;3.5$$

### Clinical outcome

The follow-up period of the study was up to 5 years, with clinical management decisions being made exclusively by the treating clinicians and not affected by the study. The primary end points measured were patient and technique survival. Patient survival was censored when there was recovery of renal function, transfer to other dialysis centers, loss to follow-up, or kidney transplant, while technique survival was censored at patient death, kidney transplant, recovery of renal function, loss to follow-up, or transfer to other dialysis centers. Secondary outcomes included the number of hospital admissions and hospital stays adjusted for the duration of follow-up, peritonitis rate, and the rate of decline in residual renal function.

### Statistical analysis

Statistical analysis was performed by SPSS for Windows software version 25.0 (IBM, Armonk, NY). The normality of data distribution was checked by the Shapiro-Wilk Test. Summary statistics were described as frequency (%) for categorical variables and mean ± SD or median (interquartile range [IQR]) for continuous variables as appropriate. Demographic and clinical data were compared between quartiles of plasma omentin-1 level by analysis of variance (ANOVA) and Chi-Square test as appropriate. The correlation between variables was explored by Spearman’s rank correlation coefficient. Survival rates were analyzed by the Kaplan–Meier survival curves. Survival analysis was further performed by the Cox proportional hazard model. Hospitalization data were analyzed by linear regression after log transformation. In either case, univariate analysis was first performed, and a multivariable regression model was then constructed by variables with *P* ≤0.1 by univariate analysis. A *P* value of < 0.05 was considered statistically significant. All probabilities were two-tailed.

## Results

We recruited 146 new PD patients. Their average age was 58.4 ± 11.8 years; 96 patients (65.8%) were men, and 90 (61.6%) had diabetes. The median vaspin level was 0.18 ng/dL (IQR 0.11 to 0.30 ng/dL). Their baseline demographic and clinical characteristics are grouped according to the plasma vaspin level quartile and summarized in Tables 1 and 2, respectively. The correlation between plasma vaspin levels and other clinical parameters, grouped according to the diabetic status, are further described in Supplementary Table 1. In essence, plasma vaspin level had modest correlations with diastolic blood pressure, E: I ratio, and carotid-radial pulse wave velocity. However, plasma vaspin level did not have a significant correlation with adipose tissue mass or other anthropometric and biochemical parameters.

**Table 1:** Baseline demographic and clinical characteristics according to the plasma vaspin level quartile

Plasma vaspin level quartile	All case	I	II	III	IV	P
No. of patients	146	36	37	37	36	
Plasma vaspin level (ng/mL)	0.54 ± 1.56	0.07 ± 0.03	0.14 ± 0.02	0.23 ± 0.05	1.72 ± 2.86	
Age (year)	58.4 ± 11.8	55.6 ± 15.1	58.0 ± 10.0	59.9 ± 10.1	60.3 ± 11.3	0.077^a^
Sex (M: F)	96:50	27:9	24:13	25:12	20:16	0.300^c^
Blood pressure (mmHg)						
Systolic	140 ± 19	142 ± 20	138 ± 17	141 ± 20	140 ± 19	0.939^a^
Diastolic	76 ± 13	81 ± 16	74 ± 13	75 ± 10	74 ± 11	0.032^a^
Primary renal disease, no. of patients (%)						0.562^c^
Diabetes mellitus	75 (51.4%)	15 (41.7%)	21 (56.8%)	17 (45.9%)	22 (61.1%)	
Hypertension	13 (8.9%)	2 (5.6%)	2 (5.4%)	7 (18.9%)	2 (5.6%)	
Glomerulonephritis	31 (21.2%)	9 (25.0%)	7 (18.9%)	7 (18.9%)	8 (22.2%)	
Polycystic kidney disease	3 (2.1%)	1 (2.8%)	0 (0.0%)	1 (2.7%)	1 (2.8%)	
Urological	6 (4.1%)	3 (8.3%)	2 (5.4%)	1 (2.7%)	0 (0.0%)	
Others	1 (0.7%)	0 (0.0%)	0 (0.0%)	1 (2.7%)	0 (0.0%)	
Unknown	12 (8.2%)	4 (11.1%)	4 (10.8%)	3 (8.1%)	1 (2.8%)	
Missing	5 (3.4%)	2 (5.6%)	1 (2.7%)	0 (0.0%)	2 (5.6%)	
Major comorbidities, no. of patients (%)						
Diabetes Mellitus	90 (61.6%)	18 (50.0%)	24 (64.9%)	23 (62.2%)	25 (69.4%)	0.125^b^
Ischemic heart disease	39 (26.7%)	13 (36.1%)	7 (18.9%)	8 (21.6%)	11 (30.6%)	0.653^b^
Cerebrovascular accident	28 (19.2%)	12 (33.3%)	5 (13.5%)	6 (16.2%)	5 (13.9%)	0.054^b^
Peripheral vascular disease	12 (8.2%)	5 (13.9%)	0 (0.0%)	3 (8.1%)	4 (11.1%)	0.991^b^
Charlson’s comorbidity scores	6.2 ± 2.5	6.2 ± 3.3	6.2 ± 2.3	6.0 ± 2.2	6.4 ± 2.3	0.835^a^

Data are compared by ^a^one-way analysis of variance for the linear association; ^b^Chi-square test for linearity trend; and ^c^Chi-square test

**Table 2:** Baseline anthropometric and biochemical characteristics according to the plasma vaspin level quartile

Plasma vaspin level quartile	All case	I	II	III	IV	P^a^
No. of patients	146	36	37	37	36	
Anthropometric measures						
body weight (kg)	66.47 ± 14.42	66.14 ± 11.71	65.36 ± 16.61	68.75 ± 15.71	65.55 ± 13.31	0.872
body mass index (kg/m2)	24.81 ± 4.20	24.88 ± 3.91	24.13 ± 4.44	25.32 ± 4.95	24.91 ± 3.39	0.684
waist circumference (cm)	88.74 ± 11.52	89.35 ± 11.87	87.89 ± 11.50	89.97 ± 12.48	87.74 ± 10.48	0.762
hip circumference (cm)	95.35 ± 9.20	96.32 ± 9.17	94.74 ± 8.94	96.04 ± 9.42	94.29 ± 9.50	0.505
waist-hip ratio	0.93 ± 0.07	0.93 ± 0.06	0.93 ± 0.06	0.93 ± 0.07	0.93 ± 0.08	0.591
mid-arm circumference (cm)	25.83 ± 3.06	26.18 ± 2.30	25.40 ± 2.85	25.83 ± 3.48	25.93 ± 2.94	0.896
triceps skin fold	9.79 ± 3.79	9.60 ± 3.87	8.87 ± 3.90	9.54 ± 3.72	11.18 ± 3.44	0.061
sub-scapular skin fold	11.29 ± 4.78	12.22 ± 4.32	10.90 ± 4.59	9.97 ± 5.10	12.11 ± 4.90	0.720
Other nutritional scores						
SGA	5.32 ± 0.87	5.27 ± 0.88	5.50 ± 0.82	5.43 ± 0.93	5.10 ± 0.85	0.486
MIS	6.7 ± 3.6	7.6 ± 3.7	5.4 ± 3.6	6.4 ± 2.5	7.4 ± 4.3	0.911
Total Kt/V	2.06 ± 0.65	2.17 ± 0.81	1.96 ± 0.71	1.95 ± 0.50	2.15 ± 0.50	0.890
Residual GFR (ml/min/1.73m^2^)	3.94 ± 2.65	4.54 ± 3.25	3.83 ± 2.75	3.65 ± 2.22	3.73 ± 2.26	0.212
Peritoneal transport characteristics						
D/P4	0.69 ± 0.12	0.68 ± 0.13	0.72 ± 0.10	0.68 ± 0.13	0.68 ± 0.13	0.593
MTAC (ml/min/1.73m2)	11.05 ± 5.07	10.55 ± 4.53	11.94 ± 4.97	10.50 ± 4.99	11.24 ± 5.82	0.899
Hemoglobin (g/dL)	8.94 ± 1.19	9.19 ± 1.29	8.82 ± 1.48	8.86 ± 0.74	8.90 ± 1.15	0.369
Albumin (g/L)	35.20 ± 4.35	35.83 ± 3.74	34.91 ± 4.85	35.24 ± 4.28	34.82 ± 4.55	0.415
HbA1c (%)	6.37 ± 1.14	6.13 ± 1.08	6.51 ± 1.12	6.35 ± 1.27	6.48 ± 1.11	0.502
Serum lipid profile						
Total cholesterol (mmol/L)	4.54 ± 1.22	4.29 ± 1.14	4.82 ± 1.21	4.50 ± 1.27	4.57 ± 1.24	0.596
Triglyceride (mmol/L)	1.54 ± 0.87	1.59 ± 0.81	1.65 ± 0.76	1.34 ± 0.52	1.60 ± 1.25	0.665
LDL cholesterol (mmol/L)	2.60 ± 1.05	2.37 ± 0.94	2.86 ± 1.07	2.53 ± 1.56	2.62 ± 0.99	0.624
HDL cholesterol (mmol/L)	1.26 ± 0.39	1.20 ± 0.37	1.21 ± 0.29	1.36 ± 0.45	1.53 ± 0.41	0.294
Serum PTH (ng/L)	58.45 ± 81.82	73.46 ± 154.79	61.59 ± 38.21	52.68 ± 29.33	46.15 ± 31.74	0.151
NPNA (g/kg/day)	1.12 ± 0.23	1.07 ± 0.24	1.15 ± 0.22	1.10 ± 0.20	1.17 ± 0.25	0.177
FEBM (%)	40.09 ± 11.56	40.95 ± 15.46	41.24 ± 10.27	39.41 ± 11.25	38.83 ± 8.27	0.383
Bioimpedance spectroscopy						
Overhydration (Litre)	4.53 ± 3.22	3.63 ± 2.03	4.62 ± 3.64	4.90 ± 3.38	4.89 ± 3.49	0.109
E:I ratio	1.02 ± 0.17	0.97 ± 0.14	1.00 ± 0.18	1.04 ± 0.20	1.05 ± 0.15	0.042
Lean tissue mass (kg)	41.04 ± 10.97	40.68 ± 9.47	40.81 ± 10.50	43.21 ± 10.35	39.25 ± 13.31	0.839
Adipose tissue mass (kg)	20.23 ± 11.18	20.70 ± 12.08	19.91 ± 11.95	20.09 ± 11.22	20.25 ± 9.93	0.897
Pulse wave velocity (cm/sec)						
Carotid-radial	10.53 ± 1.36	11.10 ± 1.43	10.37 ± 1.24	10.60 ± 1.17	10.07 ± 1.44	0.007
Carotid-femoral	11.51 ± 2.36	11.70 ± 2.38	11.40 ± 2.22	11.31 ± 2.44	11.63 ± 2.46	0.866
Serum markers of inflammation						
C-peptide (ng/mL)	9.12 ± 7.18	9.14 ± 8.52	11.23 ± 9.24	7.17 ± 4.60	8.85 ± 4.61	0.360
C-reactive protein (mg/L)	13.21 ± 30.07	21.04 ± 43.84	10.93 ± 31.27	10.24 ± 18.37	11.28 ± 21.16	0.220

*LDL *Low-density lipoprotein, *HDL *High-density lipoprotein, *PTH *Parathyroid hormone, *GFR *Glomerular filtration rate, *D/P4 *Dialysate-to-plasma creatinine concentration at 4 h, *MTAC *Mass transfer areas coefficient of creatinine, *FEBM *Fat-free edema-free body mass, *SGA S*ubjective global assessment overall score, *MIS *Malnutrition inflammation score, *E: I ratio E*xtra-cellular to intracellular volume ratio, NAPA for normalized protein nitrogen appearance. Data are compared by ^a^one-way analysis of variance for the linear association

### Insulin resistance and body adiposity

The average plasma vaspin concentrations of people with diabetes and those without were 0.68 ± 1.92 ng/mL and 0.32 ± 0.66 ng/mL, respectively (*p* = 0.110). Supplementary Table 2 lists the relationship between plasma vaspin levels and parameters of insulin resistance and body fat. In those with diabetes, there was a significant inverse correlation between plasma vaspin levels and baseline conicity index (*r*=-0.219, *p* = 0.041). In those without diabetes, a modest link was observed between plasma vaspin levels and the change in HOMA IR (*r*=-0.358, *p* = 0.048) from baseline to 24 months.

### Patient and technique survival

The average follow-up period was 47.0 ± 32.2 months. In the follow-up phase, 87 patients died, 17 were converted to long-term hemodialysis, 13 had kidney transplants, and 6 moved to different centers. The causes of death were ischemic heart diseases (23 patients), cerebrovascular accidents (9 patients), sudden cardiac arrest (5 patients), peritonitis (9 patients), non-peritonitis infections (33 patients), malignancy (3 individuals), termination of dialysis (2 patients), and other specific causes (3 patients). According to the quartiles of plasma vaspin level quartiles I to IV (from lowest to highest), the 5-year patient survival rates were 19.5%, 37.5%, 31.0%, and 36.7%, respectively (univariate Cox analysis, *p* = 0.171), and the corresponding technique survival rates were 94.1%, 95.5%, 79.9%, and 79.8%, respectively (*p* = 0.698). Vaspin level was not associated with cardiovascular survival (details not shown). The result of survival analysis remained similar when vaspin levels were adjusted to BMI or BSA (details not shown).

When analyzing the patient and technique survival according to the diabetic status, the results showed a significant interaction between the quartiles of plasma vaspin level and diabetic status (*p* < 0.001) (Supplementary Table 3), but not on technique survival (*p* = 0.834). When patient survival was analyzed according to the diabetic status, plasma vaspin level quartiles were significantly associated with the patient survival for patients without diabetes, but not with diabetic patients (Table 3). In nondiabetic patients, each higher quartile of plasma vaspin level was associated with an approximately two-fold increase in mortality risk.

**Table 3:** Multi-variable cox regression for patient survival patients with diabetes

	Univariate analysis			Multivariable analysis		
Variable	HR	95%CI	*p*-value	AHR	95%CI	*p*-value
Patients with diabetes						
Plasma vaspin level quartile	0.975	0.780-1.218	0.820	0.922	0.670-1.268	0.617
Sex^a^	0.789	0.443-1.404	0.420			
Age	1.022	0.992-1.053	0.144			
BMI	0.995	0.932-1.062	0.876			
CCI	1.179	1.044-1.332	0.008	1.132	0.903-1.420	0.283
SGA	0.564	0.361-0.881	0.012	0.614	0.332-1.172	0.139
MIS	1.079	0.990-1.175	0.083	0.892	0.754-1.054	0.180
Serum albumin	0.936	0.882-0.993	0.029	0.988	0.875-1.116	0.845
HbA1c	1.241	0.940-1.638	0.128			
LDL	0.950	0.753-1.198	0.664			
Serum CRP	0.999	0.992-1.007	0.899			
Total Kt/V	0.869	0.614-1.231	0.430			
residual GFR	0.942	0.854-1.039	0.229			
CF_PWV	0.987	0.887-1.098	0.803			
NPNA	0.373	0.146-0.953	0.039	0.461	0.108-1.967	0.295
MTAC	0.987	0.942-1.035	0.597			
Overhydration	1.055	0.964-1.154	0.248			
E: I ratio	4.273	0.872-20.939	0.073	12.088	0.558-261.806	0.112
Adipose tissue mass	1.010	0.991-1.029	0.302			
Lean tissue mass	0.974	0.950-0.999	0.040	0.988	0.936-1.042	0.650
Serum PTH	1.008	1.000-1.015	0.058	1.008	0.996-1.020	0.173
Serum C-peptide	0.976	0.938-1.016	0.236			
Patients without diabetes						
Variable	HR	95%CI	p-value	AHR	95%CI	p-value
Plasma vaspin level quartile	1.489	0.993-2.232	0.054	2.038	1.191-3.487	0.009
Sex^a^	1.157	0.486-2.755	0.742			
Age	1.105	1.056-1.156	<0.001	1.081	0.687-1.703	0.736
BMI	0.995	0.870-1.139	0.945			
CCI	1.493	1.208-1.844	<0.001	1.169	0.888-1.538	0.265
SGA	0.641	0.238-1.725	0.379			
MIS	1.148	0.941-1.402	0.174			
Serum albumin	0.990	0.914-1.073	0.812			
HbA1c	3.438	1.130-10.462	0.030	5.905	1.302-26.793	0.021
LDL	1.273	0.881-1.839	0.198			
Serum CRP	0.998	0.985-1.012	0.829			
Total Kt/V	1.266	0.416-3.859	0.678			
residual GFR	1.025	0.864-1.215	0.780			
CF_PWV	1.207	0.960-1.516	0.107			
NPNA	1.401	0.085-23.078	0.814			
MTAC	1.044	0.965-1.129	0.285			
Overhydration	1.091	0.900-1.323	0.374			
E: I ratio	62.855	1.995-1980.675	0.019	29.769	0.606-1462.659	0.088
Adipose tissue mass	1.019	0.968-1.072	0.473			
Lean tissue mass	0.979	0.939-1.020	0.312			
Serum PTH	1.003	1.000-1.005	0.018	1.005	1.002-1.008	<0.001
Serum C-peptide	0.998	0.956-1.042	0.928			

*HR *Hazards ratio, *AHR *Adjusted hazards ratio, *CI*, Confidence interval, *GFR *Glomerular filtration rate, *CCI *Charlson’s comorbidity index, *SGA *Subjective global assessment overall score, *MIS *Malnutrition inflammation score, *CR_PWV *Carotid to radial pulse wave velocity, *CF_PWV C*arotid to femoral pulse wave velocity, *BMI *Body mass index, *hsCRP *High sensitivity C-reactive protein, *MTAC *Mass transfer areas coefficient of creatinine, *NPNA *Normalized protein nitrogen appearance, E: I ratio, extra-cellular to intracellular volume ratio, *HDL *High-density lipoprotein, *LDL *Low-density lipoprotein, *PTH *Parathyroid hormone

^a^male as compared to female

### Relation with hospitalization

During the first two years after PD, there were 846 hospital admissions for a total of 6013 days. The median hospital admission rate was 1.64 episodes per year (IQR 0.85 to 3.27), and the median duration of hospital stay was 9.54 days per year (IQR 3.06 to 21.84). There was no significant correlation between the plasma vaspin level quartile and the rate of hospital admission (*r* = 0.097, *p* = 0.255) or the duration of hospitalization (*r* = 0.080, *p* = 0.346).

### Residual renal function and peritonitis

During the follow-up period, 60 patients developed anuria. The median rate of residual renal function decline was − 1.22 ml/min/1.73m^2^ per year (IQR − 2.19 to -0.52). There was no significant correlation between the rate of residual GFR decline and plasma vaspin level (*r* = 0.106, *p* = 0.266). The 5-year anuria-free survival rates of plasma vaspin level quartiles, from the lowest to highest, were 29.3%, 11.8%, 23.6%, and 36.2% respectively (univariate Cox analysis, *p* = 0.730).

During the follow-up period, 86 patients had 185 peritonitis episodes. The overall peritonitis rate was 0.43 episodes per patient year. There was no significant correlation between the peritonitis rate and plasma vaspin level (*r* = 0.042, *p* = 0.624). The 5-year peritonitis-free survival rates of plasma vaspin level quartiles, from the lowest to highest, were 23.4%, 35.9%, 39.9%, and 18.4% respectively (univariate Cox analysis, *p* = 0.647).

## Discussion

The results of our study suggest that plasma vaspin level quartile is a significant predictor of patient survival for non-diabetic PD patients. Additionally, there is a modest correlation between plasma vaspin level and the conicity index, which is an indicator of central obesity, as well as the subsequent change in insulin resistance. However, no significant correlation was found between plasma vaspin level quartile and any other anthropometric or biochemical parameter, including adipose tissue mass, technique survival, rate of hospitalization, peritonitis rate, or the rate of residual renal function decline. These findings emphasize the importance of plasma vaspin level quartile as a potential predictor of patient survival and suggest that further research should focus on elucidating the mechanisms underlying these correlations.

Our findings were in line with previous studies [21, 22]. We found the plasma vaspin levels in our PD patients to be comparable to the hemodialysis patients [22], but much lower than the pre-dialysis CKD patients [21]. Demir et al. [21] observed an elevated vaspin level with a higher HOMA-IR index in pre-dialysis CKD patients, yet they found no correlation between the two due to the limited sample size. Though we also did not find any association between the serum vaspin level and the HOMA-IR baseline, a modest inverse correlation between the two was seen with regard to the subsequent change in HOMA-IR, indicating the potential role of vaspin in the regulation of insulin sensitivity [19, 20]. Our results contrast with the report of Inoue et al. [22] which found a correlation between vaspin and creatinine, as well as triglyceride levels in hemodialysis patients. This difference may be attributed to the difference in dialysis modality, glucose exposure, and the higher waist circumference in our study population.

The findings of our study, which employed a larger sample size than previous studies [21, 22], suggest that higher plasma vaspin levels may be associated with mortality risk in non-diabetic patients. This is in contrast to the previous reports [41, 42], which did not find any relation between vaspin level and systemic inflammation. There was also modest correlation between plasma vaspin levels and changes in HOMA-IR and serum insulin levels in non-diabetic patients, implying that vaspin may be involved in the progression of metabolic disturbances after PD. However, the magnitude of this involvement may not be sufficient to explain the association between vaspin level quartile and survival rate. In addition, the association between vaspin level and survival rate was absent in diabetic patients, possibly due to their advanced state of metabolic disturbance, in which vaspin no longer has a meaningful biological role.

In addition to the reasonable sample size, there were several strengths of our study. First, our study is the first in this field that was dedicated to PD patients. Second, we have a comprehensive assessment of the patients’ biochemical profile, nutritional status, peritoneal transport, body composition, and vascular stiffness. Notably, we performed a detailed serial assessment for the change in insulin profile, which, to the best of our knowledge, has not been reported in other studies.

However, there were several important limitations of our present study. First, vaspin levels were measured only at the initiation of PD. With the observation that baseline vaspin level correlated with the change in insulin resistance, and since weight gain is exceedingly common in new PD patients [10], it would be interesting to determine if there is any concomitant change in vaspin level or body composition. Second, we did not look into the possible mechanisms for the relation between vaspin and insulin resistance, which has been the focus of several other reports [43, 44]. Several nutritional parameters were added simultaneously in our Cox regression models, which may create a problem of collinearity. However, only significant internal correlations existed between Charlson’s score, MIS and SGA scores, and the result of Cox regression remained similar when these parameters were used separately for the creation of Cox models (details not show). On the other hand, the assessment of lean body mass (LBM) may not be accurate. According to a previous study that compared LBM estimated from anthropometry and dual-energy X-ray absorptiometry (DEXA), the accuracy of the former was poor [45]. A similar problem may also exist when evaluating the adipose tissue mass by anthropometric measurements, which may explain the seemingly insignificant difference of adipose tissue mass between vaspin level quartiles.

In summary, our study showed that the plasma vaspin level quartile had a significant association with patient survival in non-diabetic PD patients. Baseline plasma vaspin level also had a modest inverse correlation with the subsequent change in the severity of insulin resistance, but the exact biological role of vaspin deserves further studies.

## Supplementary Information

**Additional file 1: Supplementary Table 1.** Correlation between plasma vaspin level and baseline clinical and biochemical parameters according to diabetic status. PDE, peritoneal dialysis effluent; LDL, low-density lipoprotein; HDL, high-density lipoprotein; PTH, parathyroid hormone; SGA, subjective global assessment overall score; MIS, malnutrition inflammation score; E: I ratio, extra-cellular to intracellular volume ratio;  D/P4, dialysate-to-plasma creatinine concentration at 4 hours; MTAC, mass transfer areas coefficient of creatinine; GFR, glomerular filtration rate; FEBM, fat-free edema-free body mass, NAPA for normalized protein nitrogen appearance; PWV, pulse wave velocity. Data are compared by Spearman’s rank correlation coefficient. **Supplementary Table 2.** Correlation between plasma vaspin level and insulin resistance. HbA1C, Hemoglobin A1C; HOMA, Homeostatic Model Assessment; IR, Insulin resistance; 2B, beta 2 cell function. Data are compared by Spearman’s rank correlation coefficient. **Supplementary Table 3.** Cox regression analysis for patient survival (all patients). HR, hazards ratio; AHR, adjusted hazards ratio; CI, confidence interval; GFR, glomerular filtration rate; CCI, Charlson’s comorbidity index; SGA, subjective global assessment overall score; MIS, malnutrition inflammation score; CR_PWV, carotid to radial pulse wave velocity; CF_PWV, carotid to femoral pulse wave velocity, BMI, body mass index; hsCRP, high sensitivity C-reactive protein; MTAC, mass transfer areas coefficient of creatinine; NPNA, normalized protein nitrogen appearance; E: I ratio, extra-cellular to intracellular volume ratio; HDL, high-density lipoprotein; LDL, Low-density lipoprotein; PTH, parathyroid hormone. *interaction between plasma vaspin level quartile and diabetic status. **male as compared to female

## Data Availability

The datasets used and/or analysed during the current study available from the corresponding author on reasonable request.

## Abbreviations

ADA: American Diabetes Association
AHR: Adjusted hazards ratio
ANOVA: Analysis of variance
ATM: Adipose tissue mass
BMI: Body mass index
BSA: Body surface area
CCI: Charlson’s comorbidity index
CI: Confidence interval
CKD: Chronic kidney disease
CRP: C-reactive protein
CVD: Cardiovascular disease
D/P: Dialysate-to-plasma creatinine ratio
E:I: Extracellular-to-intracellular volume
FEBM: Fat-free edema-free body mass
GFR: Glomerular filtration rate
HDL: High-density lipoprotein
HR: Hazards ratio
IQR: Interquartile range
LDL: Low-density lipoprotein
LTM: Lean tissue mass
MIS: Malnutrition–Inflammation Score
MTAC: Mass transfer area coefficients
NPNA: Normalized protein nitrogen appearance
OH: Volume of over-hydration
PD: Peritoneal dialysis
PET: Peritoneal equilibration test
PTH: Parathyroid hormone
PWV: Pulse wave velocity
SGA: Subjective Global Assessment
WHR: Waist-hip ratio
