# Supplementary material for: Plasma vaspin levels and clinical outcome in incident peritoneal dialysis patients
Source: BMC Nephrol. 2023 Jul 12;24:206. doi: 10.1186/s12882-023-03259-2 (PMC10337148; doi:10.1186/s12882-023-03259-2)
Supplement: Supplementary file 1 — Additional file 1: Supplementary Table 1. Correlation between plasma vaspin level and baseline clinical and biochemical parameters according to diabetic status. PDE, peritoneal dialysis effluent; LDL, low-density lipoprotein; HDL, high-density lipoprotein; PTH, parathyroid hormone; SGA, subjective global assessment overall score; MIS, malnutrition inflammation score; E: I ratio, extra-cellular to intracellular volume ratio; D/P4, dialysate-to-plasma creatinine concentration at 4 hours; MTAC, mass transfer areas coefficient of creatinine; GFR, glomerular filtration rate; FEBM, fat-free edema-free body mass, NAPA for normalized protein nitrogen appearance; PWV, pulse wave velocity. Data are compared by Spearman’s rank correlation coefficient. Supplementary Table 2. Correlation between plasma vaspin level and insulin resistance. HbA1C, Hemoglobin A1C; HOMA, Homeostatic Model Assessment; IR, Insulin resistance; 2B, beta 2 cell function. Data are compared by Spearman’s rank correlation coefficient. Supplementary Table 3. Cox regression analysis for patient survival (all patients). HR, hazards ratio; AHR, adjusted hazards ratio; CI, confidence interval; GFR, glomerular filtration rate; CCI, Charlson’s comorbidity index; SGA, subjective global assessment overall score; MIS, malnutrition inflammation score; CR_PWV, carotid to radial pulse wave velocity; CF_PWV, carotid to femoral pulse wave velocity, BMI, body mass index; hsCRP, high sensitivity C-reactive protein; MTAC, mass transfer areas coefficient of creatinine; NPNA, normalized protein nitrogen appearance; E: I ratio, extra-cellular to intracellular volume ratio; HDL, high-density lipoprotein; LDL, Low-density lipoprotein; PTH, parathyroid hormone. *interaction between plasma vaspin level quartile and diabetic status. **male as compared to female [file 12882_2023_3259_MOESM1_ESM.pdf]

STROBE Statement—checklist of items that should be included in reports of observational studies

	Item No.	Recommendation	Page No.	Relevant text from manuscript
**Title and abstract**	1	(*a*) Indicate the study's design with a commonly used term in the title or the abstract	1	
		(*b*) Provide in the abstract an informative and balanced summary of what was done and what was found	3	Abstract
**Introduction**				
Background/rationale	2	Explain the scientific background and rationale for the investigation being reported	4,5	Introduction
Objectives	3	State specific objectives, including any prespecified hypotheses	4,5	
**Methods**				
Study design	4	Present key elements of study design early in the paper	6,7	Patients and Methods
Setting	5	Describe the setting, locations, and relevant dates, including periods of recruitment, exposure, follow-up, and data collection	7,8	
Participants	6	(*a*) *Cohort study*—Give the eligibility criteria, and the sources and methods of selection of participants. Describe methods of follow-up<br>*Case-control study*—Give the eligibility criteria, and the sources and methods of case ascertainment and control selection. Give the rationale for the choice of cases and controls<br>*Cross-sectional study*—Give the eligibility criteria, and the sources and methods of selection of participants	7,8	Patients and Methods
		(*b*) *Cohort study*—For matched studies, give matching criteria and number of exposed and unexposed<br>*Case-control study*—For matched studies, give matching criteria and the number of controls per case		
Variables	7	Clearly define all outcomes, exposures, predictors, potential confounders, and effect modifiers. Give diagnostic criteria, if applicable	9	Patients and Methods
Data sources/ measurement	8*	For each variable of interest, give sources of data and details of methods of assessment (measurement). Describe comparability of assessment methods if there is more than one group	6-8	Patients and Methods
Bias	9	Describe any efforts to address potential sources of bias		
Study size	10	Explain how the study size was arrived at	6	

Continued on next page

Quantitative variables	11	Explain how quantitative variables were handled in the analyses. If applicable, describe which groupings were chosen and why	9	
Statistical methods	12	(*a*) Describe all statistical methods, including those used to control for confounding	9	Statistical Method
		(*b*) Describe any methods used to examine subgroups and interactions		Statistical Method
		(*c*) Explain how missing data were addressed	not applicable	
		(*d*) *Cohort study*—If applicable, explain how loss to follow-up was addressed<br>*Case-control study*—If applicable, explain how matching of cases and controls was addressed<br>*Cross-sectional study*—If applicable, describe analytical methods taking account of sampling strategy	9	
		(*e*) Describe any sensitivity analyses		
**Results**				
Participants	13*	(a) Report numbers of individuals at each stage of study—eg numbers potentially eligible, examined for eligibility, confirmed eligible, included in the study, completing follow-up, and analysed	10	
		(b) Give reasons for non-participation at each stage	10	
		(c) Consider use of a flow diagram	not applicable	
Descriptive data	14*	(a) Give characteristics of study participants (eg demographic, clinical, social) and information on exposures and potential confounders	10	Results & Table 1
		(b) Indicate number of participants with missing data for each variable of interest	10	
		(c) *Cohort study*—Summarise follow-up time (eg, average and total amount)	10-12	
Outcome data	15*	*Cohort study*—Report numbers of outcome events or summary measures over time	10-12	Results & Tables 2&3
		Case-control study—Report numbers in each exposure category, or summary measures of exposure		
		Cross-sectional study—Report numbers of outcome events or summary measures		
Main results	16	(*a*) Give unadjusted estimates and, if applicable, confounder-adjusted estimates and their precision (eg, 95% confidence interval). Make clear which confounders were adjusted for and why they were included	11-12	Results & Table 4
		(*b*) Report category boundaries when continuous variables were categorized		
		(*c*) If relevant, consider translating estimates of relative risk into absolute risk for a meaningful time period		

Continued on next page

Other analyses	17	Report other analyses done—eg analyses of subgroups and interactions, and sensitivity analyses	11-12	Results
**Discussion**				
Key results	18	Summarise key results with reference to study objectives	13-15	Discussion
Limitations	19	Discuss limitations of the study, taking into account sources of potential bias or imprecision. Discuss both direction and magnitude of any potential bias	14-15	Discussion
Interpretation	20	Give a cautious overall interpretation of results considering objectives, limitations, multiplicity of analyses, results from similar studies, and other relevant evidence	15	Conclusion
Generalisability	21	Discuss the generalisability (external validity) of the study results	14-15	Discussion & Conclusion
**Other information**				
Funding	22	Give the source of funding and the role of the funders for the present study and, if applicable, for the original study on which the present article is based	29	Funding & Disclosure

*Give information separately for cases and controls in case-control studies and, if applicable, for exposed and unexposed groups in cohort and cross-sectional studies.

Note: An Explanation and Elaboration article discusses each checklist item and gives methodological background and published examples of transparent reporting. The STROBE checklist is best used in conjunction with this article (freely available on the Web sites of PLoS Medicine at http://www.plosmedicine.org/, Annals of Internal Medicine at http://www.annals.org/, and Epidemiology at http://www.epidem.com/). Information on the STROBE Initiative is available at www.strobe-statement.org.
